# Supplementary material for: Controlled Release of Chlorogenic Acid from Polyvinyl Alcohol/Poly(γ-Glutamic Acid) Blended Electrospun Nanofiber Mats with Potential Applications in Diabetic Foot Treatment
Source: Polymers (Basel). 2021 Aug 31;13(17):2943. doi: 10.3390/polym13172943 (PMC8434031; doi:10.3390/polym13172943)
Supplement: Supplementary file 1 [file polymers-13-02943-s001.zip › polymers-1312890-supplementary.pdf]

Supporting information

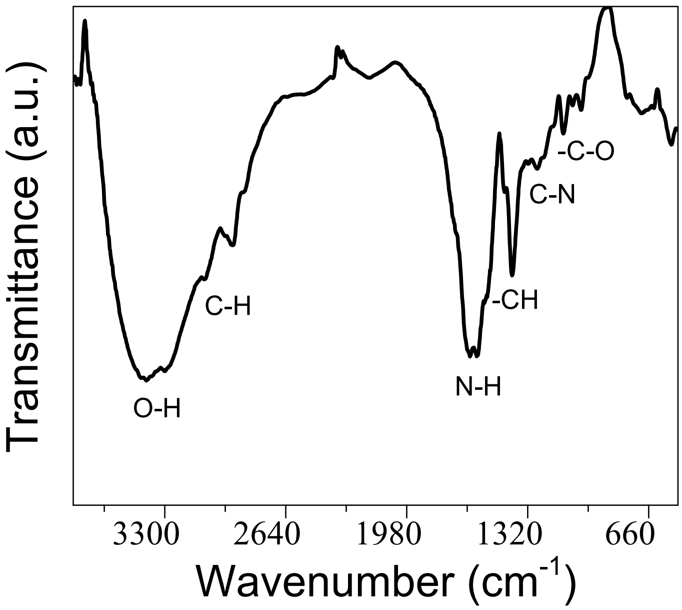

Figure S1. FT-IR spectra of γ-PGA.

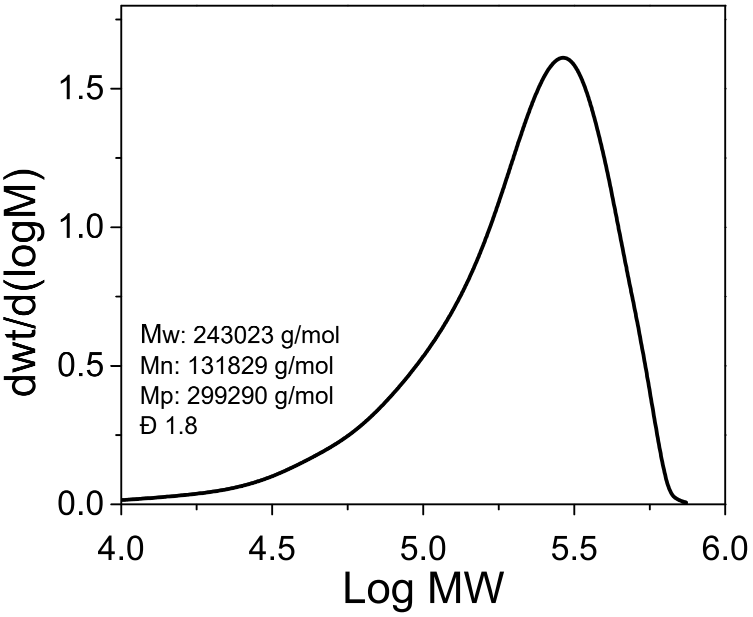

Figure S2. GPC Chromatogram of γ-PGA.

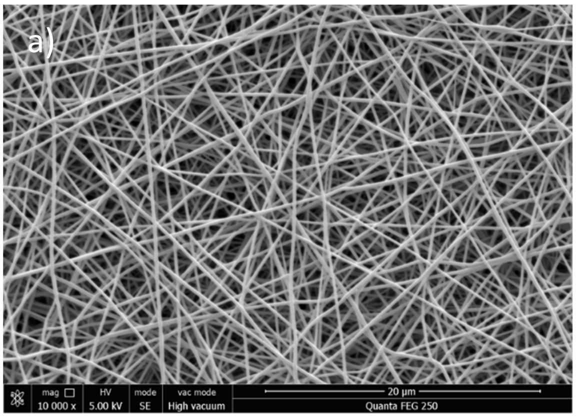

b)

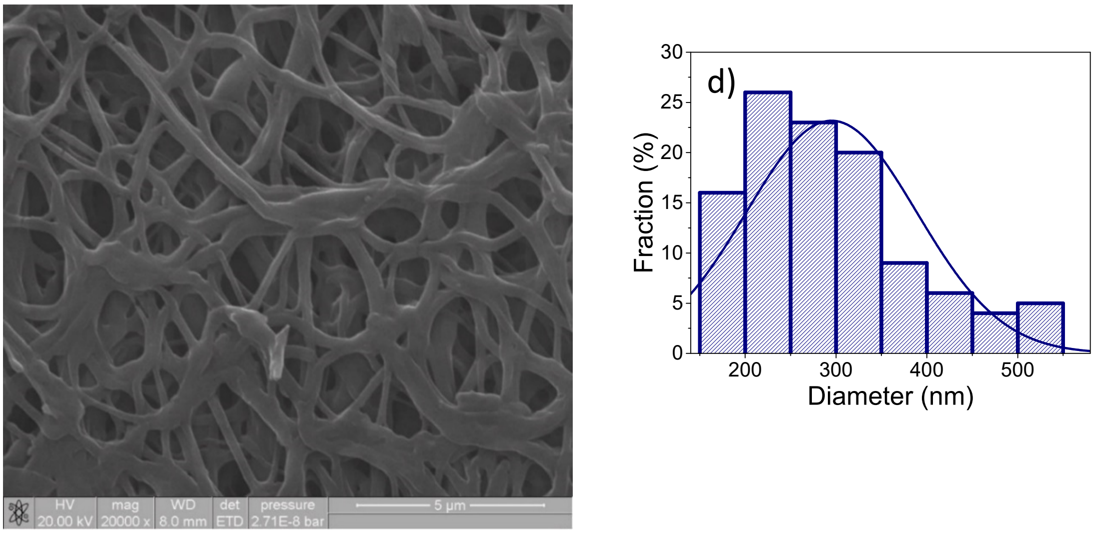

Figure S3. SEM micrographs of The PVA nanofiber, obtains by electrospun with and without crosslinking (a) and c), respectively. Diameter size distribution for nanofiber with and without crosslinking (b), d), respectively.

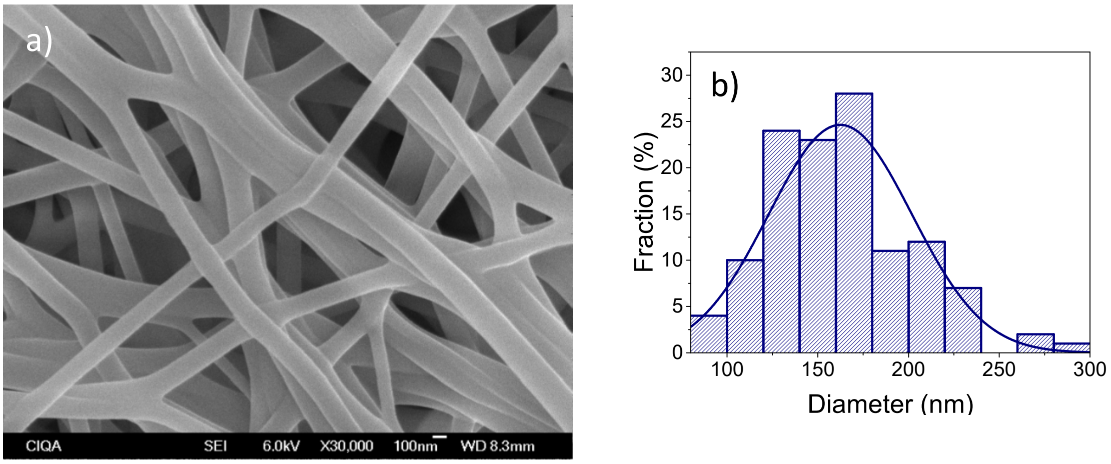

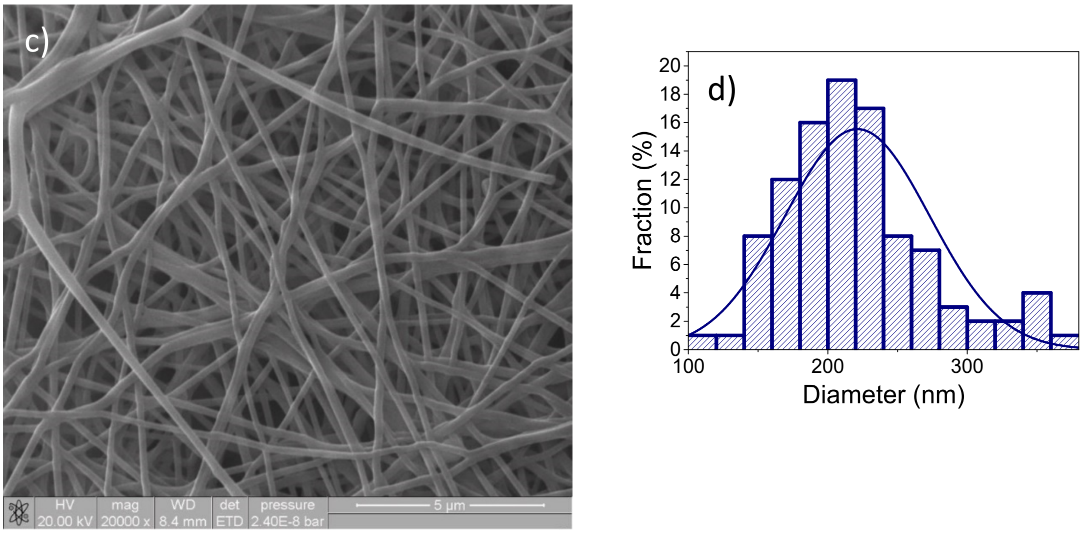

Figure S4. SEM micrographs of The PVA/γ-PGA, 5 % nanofiber, obtains by electrospun with and without crosslinking (a) and c), respectively. Diameter size distribution for nanofiber with and without crosslinking (b), d), respectively.

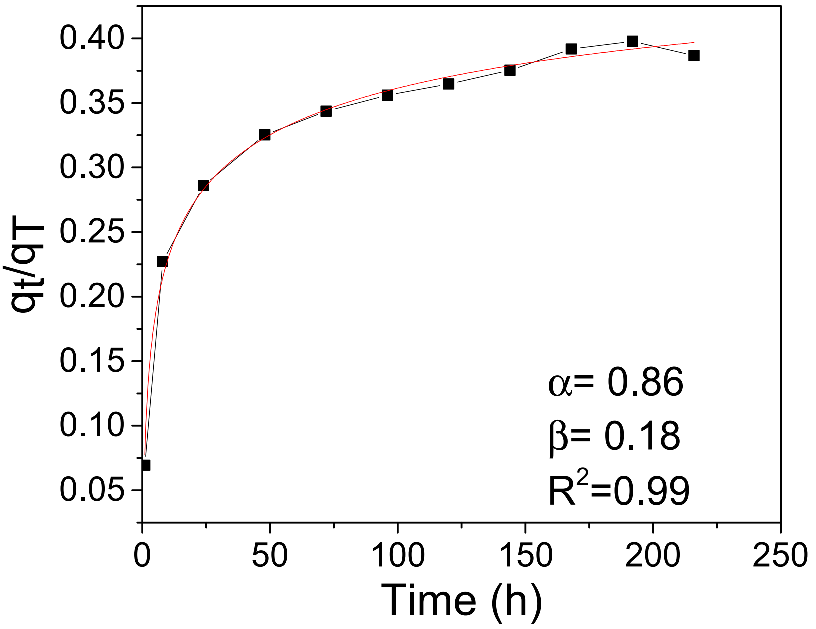

Figure s5. Weibull model for PVA.

Table S1. Average pore size for PVA, PVA/γ-PGA 5% and PVA/γ-PGA 10 %.

Sample	Average (nm)
**PVA/γ-PGA 10**	299 ± 118
**PVA/γ-PGA 5**	439 ± 168
**PVA**	723 ± 244
